# Non‐surgical management of posterior tibial tendon dysfunction‐ a UK survey

**DOI:** 10.1002/jfa2.12033

**Published:** 2024-06-19

**Authors:** Alison Miller, Toby Smith, Michael R. Backhouse

**Affiliations:** ^1^ Physiotherapy Department Heartlands Hospital University Hospitals Birmingham Birmingham UK; ^2^ Warwick Clinical Trials Unit University of Warwick Coventry UK

**Keywords:** flatfoot, national survey, posterior tibial tendon dysfunction, PTTD, tibialis posterior

## Abstract

**Background:**

Posterior Tibial Tendon Dysfunction (PTTD) is commonly seen within musculoskeletal care. The condition's prevalence and management is poorly understood. This study aims to demonstrate current practice by multi‐professional clinicians across the United Kingdom within the National Health Service.

**Methods:**

A national (UK) cross‐sectional online survey was conducted among multi‐professionals who treat PTTD within their NHS practice. The survey covered assessment, management and evaluation. This was shared via social media and professional groups.

**Results:**

Two hundred thirteen surveys were completed, with 153 matching the eligibility criteria. The main respondents were Physiotherapists (48%) and Podiatrists (38%). Ultrasound scanning was used most frequently when considering initial imaging (67%). Many different treatment modalities were used, but a core set of education/advice, foot orthoses, and foot specific as well as general exercise were most commonly chosen. Outcome measures routinely used were pain scale (96/269) and single leg heel raise (84/269), but patient reported outcome measures were not routinely used. The most frequent reason to escalate care was failure to manage symptoms with conservative management (106/123; 86.2%), followed by fixed deformity (10/123; 8.2%).

**Conclusions:**

This survey provides evidence on current non‐surgical management for PTTD from UK NHS practice. It provides a valuable marker for clinicians to use to compare their own practice and can be used in further research as a comparator.

## 1 INTRODUCTION

The tibialis posterior muscle sits within the deep posterior compartment of the leg. It acts to stabilise the medial longitudinal arch and to invert and plantarflex foot [1]. Posterior Tibial Tendon dysfunction (PTTD) typically presents as pain on the medial aspect of the ankle/foot with or without progressive flattening of the medial arch [2]. It is a progressive and disabling condition leading to impaired mobility and function [2, 3]. The prevalence of this condition is poorly understood with current data suggesting a prevalence of PTTD being 3.3% of women over 40 in the UK. However, this has previously been suggested as an underestimation [3, 4]. Early intervention has been suggested to limit the progression of PTTD, with conservative (non‐surgical) treatments advised as the initial approach for managing these people [2]. However, there is little research to support which treatment is most effective [2, 3].

People with PTTD may consult a wide range of health professionals during their patient‐journey [5]. This can create significant burden both for patients and health services, with patients undergoing a variety of examinations, investigations and treatments. The evidence base is scant, meaning clinicians remain unsure which treatment approaches are best to manage their patients [3, 5, 6]. Furthermore, there are no current best practice guidelines to inform management decisions and enable audit. In the absence of evidence‐based guidelines, clinicians seeking to evaluate their service may opt to benchmark their service against others, as is often advocated within quality improvement literature [7, 8]. While definitions of this process vary, it is essentially a process of peer comparison that identifies and reduces unwarranted variation [8, 9, 10]. To enable this, it is first necessary to describe contemporary practice.

A recent James Lind Alliance priority setting partnership highlighted the need for research into the management of people with PTTD [11]. This survey begins to address that need. We aimed to determine what the current practices are across multi‐professional teams within the NHS in the UK for people with PTTD. We hope this will help inform the direction and design of further research into non‐surgical management of early PTTD.

## 2 METHODS

### 2.1 Design

This was a national (UK‐based), cross‐sectional online survey delivered through Qualtrics (Qualtrics). The web‐based questionnaire was designed to capture data describing current practice of health professionals who treat PTTD as part of their NHS practice as previous surveys have shown differences in practice between the NHS and private sector. The survey had open‐ and closed‐questions. It was piloted amongst clinicians, including physiotherapists and podiatrists prior to distribution. No personally identifiable data were collected.

### 2.2 Participants and approach

The survey's target population was health professionals who treat people with PTTD as part of their NHS practice. These included orthopaedic surgeons, physiotherapists, podiatrists and GPs via membership of professional bodies and special interest groups which agreed to distribute the survey to their membership. These included the Association of Foot and Ankle Physiotherapists, Royal College of Podiatrists, MSK:UK and Heart of England Foot and Ankle Surgeons. The survey was also shared on social media via X where it was anticipated that this would be re‐shared to provide a wider catchment of respondents through a snowballing sampling strategy.

The survey was open for a 6‐week period (23 October 2023 to 04 December 2023). This timeframe was selected based on similar studies and to allow sufficient time for delays in publishing and distribution by national associations [12, 13, 14].

### 2.3 Consent and data collection

When health professionals accessed the first page of the survey, they were provided with a written introduction to the survey. They had the opportunity to read the embedded Participant Information Leaflet. Consent for the survey was then recorded via a tick‐box within the survey.

The survey consisted of 14 questions collecting data on respondent characteristics, where they were based in the UK and which field of work they were within, the number of patients they see with PTTD, imaging use, treatment modality use and onward referrals. Not all questions were required to be answered to complete the survey. A copy of the survey is presented as Supporting Information S1.

### 2.4 Sample size

No a priori sample size was stipulated as there is no consensus over the optimal sample size for this type of online survey. The survey was therefore limited by time, being open for 6 weeks. This was considered sufficient to obtain a large, representative sample without creating respondent fatigue.

### 2.5 Data analysis

The analysis of the data was performed through the Qualtrics online platform and Excel. Data were analysed using descriptive statistics (mean/standard deviation for continuous data, frequency/percentages for categorical data) and presented both in tables and graphically. Further disaggregation of responses relating to clinical practice was conducted for physiotherapists and podiatrists as they were the largest groups of respondents.

## 3 RESULTS

### 3.1 Respondent characteristics

A total of 213 responses were recorded of which 153 met the eligibility criteria. Others were excluded for not seeing the patients within the NHS. Table 1 illustrates the characteristics of the survey respondents.

**TABLE 1:** Characteristics of respondents.

	*n*	%
Profession		
General practitioner	4/150	3%
Orthopaedic surgeon	10/150	7%
Physiotherapist	72/150	48%
Podiatrist	57/150	38%
Podiatric surgeon	4/150	3%
Rheumatologist	0/150	0%
Other‐ please state	3/150	2%
Setting		
Community	36/143	25%
Primary care	40/143	28%
Secondary care	50/143	35%
Tertiary centre	9/143	6%
Other‐ please state	8/143	6%
Main clinical service		
First contact practitioner	17/145	12%
General practice	1/145	1%
Integrated musculoskeletal	21/145	14%
Orthopaedic foot and ankle surgery	16/145	11%
Physiotherapy	43/145	30%
Podiatry	36/145	25%
Podiatric surgery	4/145	3%
Rheumatology	1/145	1%
Other‐ please state	6/145	4%

The majority of respondents were physiotherapists (72/150; 48%) or podiatrists (57/150; 38%) working across a range of NHS settings and clinical services. There was a moderate, even split between community (36/143; 25%), primary (40/143; 28%) and secondary care (50/143; 35%). The majority of respondents were based in England (122/139; 88%), with smaller numbers from Scotland (9/139; 6%), Northern Ireland (5/139; 4%) and Wales (3/139; 2%).

**FIGURE 1:**
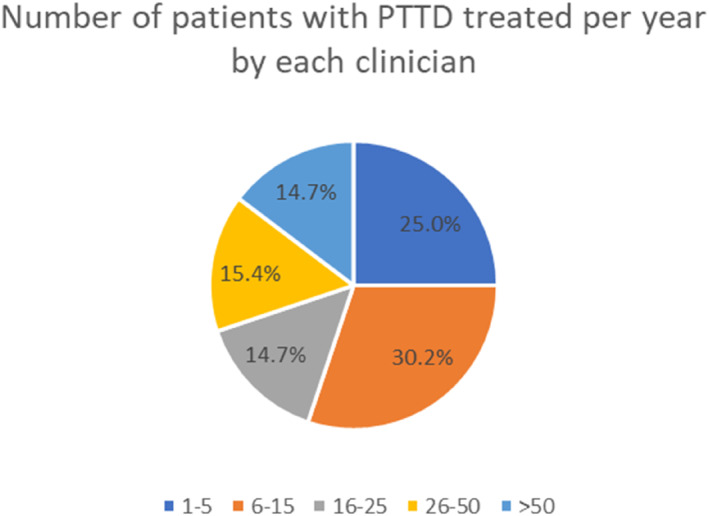
Pie chart demonstrating the estimated number of people seen by each clinician with PTTD per year. PTTD, posterior tibial tendon dysfunction.

Respondents reported that the number of patients they see with PTTD can vary (Figure 1). Over 30% of respondents (41/136) reported seeing more than one patient every 2 weeks within their caseload.

### 3.2 Imaging

**FIGURE 2:**
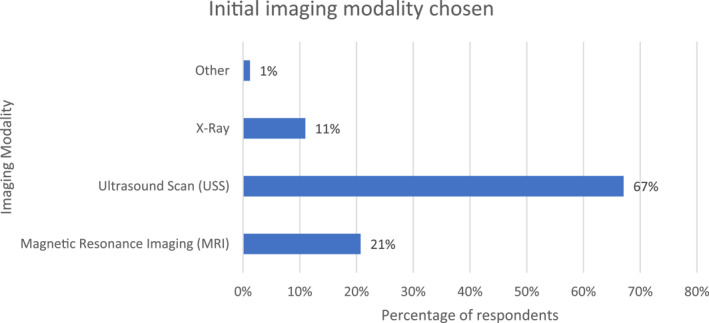
Bar chart demonstrating the initial imaging modality chosen.

Sixty‐three percent (85/136) of respondents reported that they would use imaging for these patients. Of this, the most common first‐line imaging modality was ultrasound scan (55/82; 67%) (Figure 2).

**FIGURE 3:**
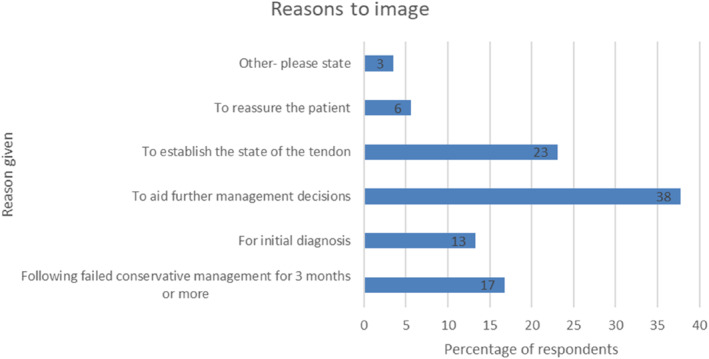
Bar chart demonstrating the reasons to image given by respondents.

A range of reasons were given as to what would prompt the clinician to request imaging (Figure 3). The principal reason was to aid further management decisions (54/143; 38%), followed by a desire to establish the state of the tendon (33/143; 23%). Other reasons identified by different respondents included

- Differential diagnosis.
- If referring onto orthopaedics as a baseline.
- To check for lateral impingement signs and deltoid/spring ligament involvement.
- Tertiary centre so often already imaged but would help with management. Decisions and to see state of tendon and rule out other differential diagnoses.

### 3.3 Treatment modalities

**FIGURE 4:**
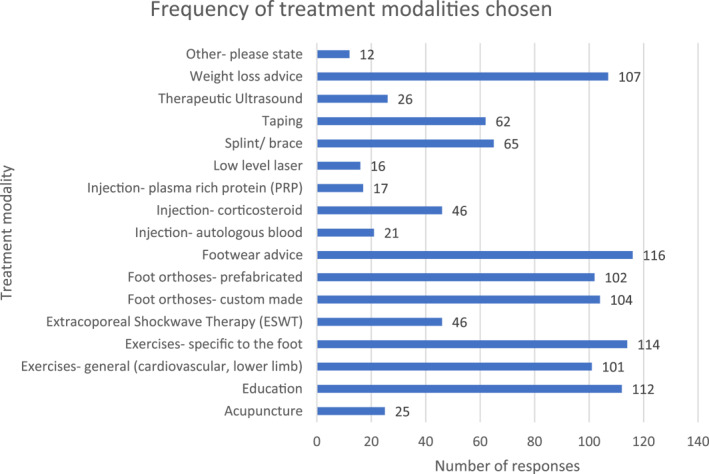
Bar chart demonstrating treatment modalities chosen.

**FIGURE 5:**
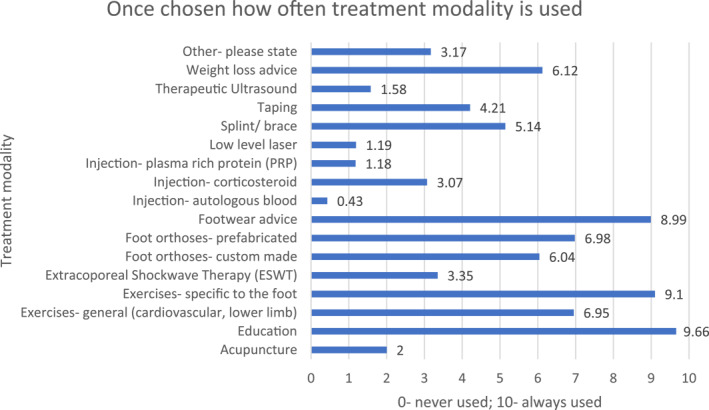
Bar chart demonstrating how often treatment modalities are used.

As demonstrated in Figure 4, the most commonly chosen treatment modalities were footwear advice, weight loss advice, foot exercises, education, foot orthoses (both prefabricated and custom made) and general exercise. Of the treatment modalities chosen, the most often used were education, footwear advice and foot exercises (Figure 5).

**FIGURE 6:**
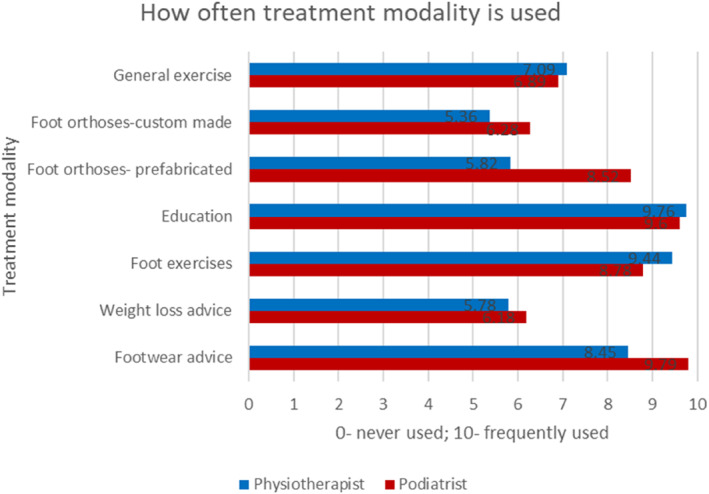
Demonstrates the use of the most common treatment modalities by Podiatrists and Physiotherapist.

**FIGURE 7:**
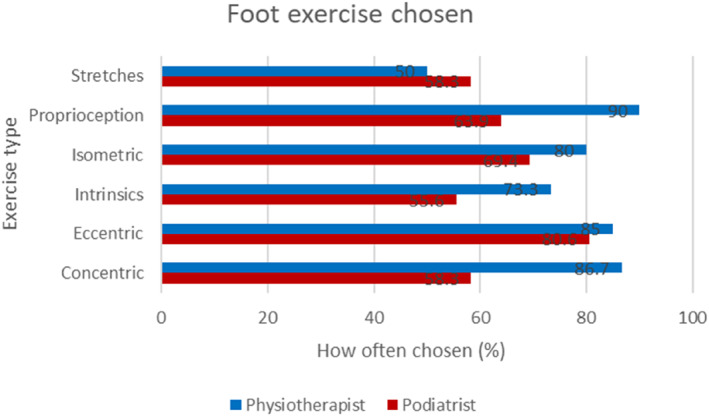
Bar chart to demonstrate the comparison between Physiotherapists and Podiatrists on foot exercise prescription.

**FIGURE 8:**
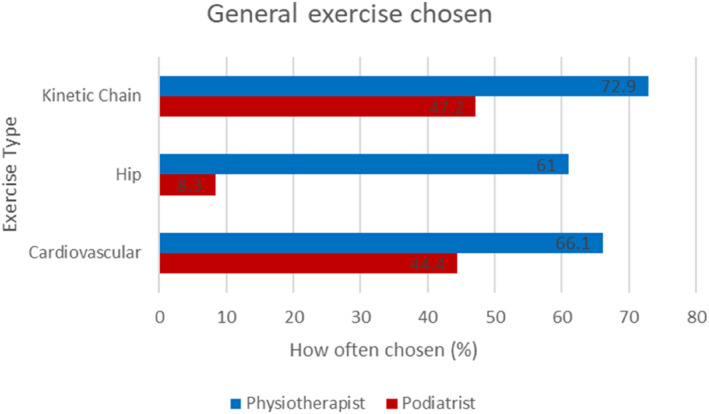
Bar chart to demonstrate the comparison between Physiotherapists and Podiatrists on general exercise prescription.

As the largest groups of respondents were physiotherapists and podiatrists, we performed subgroup analyses to evaluate potential differential treatment modalities use between the professional groups (Figures 6, 7, 8). The results demonstrate that foot orthoses and footwear advice are used more often by Podiatrists than Physiotherapists, whereas Physiotherapists prescribe more exercises. This is particularly evident when considering general exercise where there is a stark difference between Physiotherapist and Podiatrist where Kinetic chain (72.9% vs. 47.2%), hip (61% vs. 8.3%) and cardiovascular exercise (66.1% vs. 44.4%) differ (Figure 8).

### 3.4 Outcome measures

**FIGURE 9:**
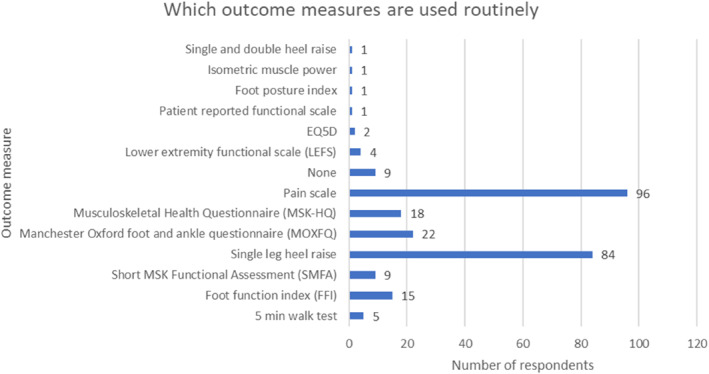
Bar chart demonstrating which outcome measures are regularly used.

Health professionals reported using multiple outcome measures for this population and were able to choose multiple options. The most frequently used routine outcome measures were pain scale (96/269) and single leg heel raise (84/269). Least commonly used outcome measures were the 5‐m walk test (5/269) and the Short MSK Functional Assessment (9/269). Interestingly, seven percent of respondents chose no routine, specific outcome measure (Figure 9). Single and double heel raise, isometric muscle power, Foot Posture Index, Patient Reported Functional Scale, EQ‐5D and Lower Extremity Functional Scale were all given in stated response to ‘other’ categories.

### 3.5 Onward referral

**FIGURE 10:**
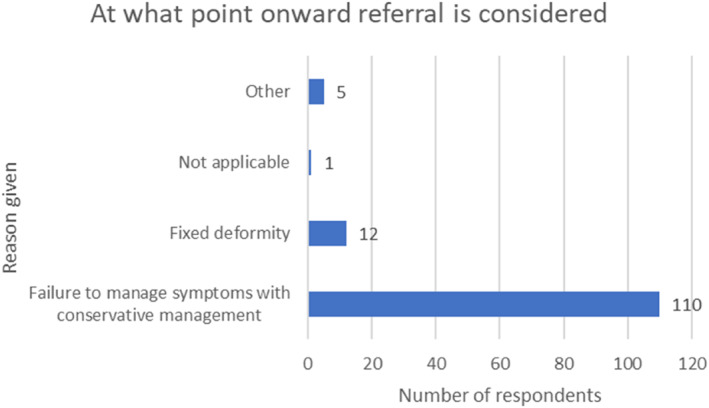
Bar chart demonstrating the reason given for onward referral.

When considering onward referral, respondents reported the most frequent reason to escalate care was failure to manage symptoms with conservative management (106/123%; 86.2%), followed by fixed deformity (10/123; 8.2%). As illustrated in Figure 10, other reasons stated were pain management, if surgery warranted for a tear, and patient choice.

## 4 DISCUSSION

This survey has demonstrated a large response from multi‐professional groups who treat PTTD within their NHS practice. These represent community, primary, secondary and tertiary centres across the NHS. The responses indicate that PTTD is seen by some clinicians on a regular basis, with over 30% of respondents seeing patients with PTTD more than fortnightly.

Imaging modalities were reported to be used by 63% of respondents. Responses indicate USS is most commonly chosen as first line diagnostic imaging modality. This is in‐line with recommendations made by the European Society of Musculoskeletal Radiology as USS being the first choice for foot and ankle tendinopathies [15, 16]. The principal reason for the consideration of imaging was to aid further management decisions, followed by establishing the state of the tendon. USS is commonly used within the foot and ankle as the structures being examined are superficial, making them more amenable to this modality. It can also provide dynamic testing of structures enabling the structure, in this case the tendon, to be assessed when it is moved [17]. For tibialis posterior tendinopathy, USS has been shown to have a high sensitivity and specificity [18]. This demonstrates that in clinical practice, USS is used as the first imaging modality when required, which is consistent with the best practice for PTTD.

The most frequently used treatment modalities were both education/advice and exercise‐based interventions with orthotics across the professional groups. This corresponds with previous research into PTTD management [3, 19]. As expected, physiotherapists reported more frequent prescription of a variety of exercise programmes considering of general and foot‐specific exercises. In contrast, podiatrists most commonly reported using orthotics (custom and prefabricated). This is in‐line with literature demonstrating foot orthosis prescription by podiatrists within the UK is used 47% of the time versus 36% for custom‐made orthotics for PTTD [20]. It important to note that all treatment modalities offered within the survey were chosen by respondents. This indicates that clinicians may try many differing treatment options. This potentially reflects the lack of guidance on how to manage this condition. The results of this survey will help to guide clinical practice to the core set of treatment modalities and help to inform future research into this area to test their effectiveness.

Our survey demonstrated that the most commonly used outcome measures were pain scale and single leg heel raise. The literature presents a wide range of potential outcome measures for this population [3, 19, 21]. There is no consistently used measure nor recommendation on what should be used, although single leg heel raise has demonstrated the best inter‐rater reliability and best comparison to USS findings [21]. Pain scale and single leg heel raise are both quick and easy to use within clinical practice, which may be an important reason why these are frequently chosen. Patient reported outcome measures were not often chosen despite recommendations of their use where possible [22, 23]. The OMERACT core outcome set for foot and ankle disorders is currently being undertaken [24]. It is hoped that this may provide recommendations on what should be measured in the management of PTTD.

This study presents with both strengths and limitations. As a strength, this study provides novel insights into the care of people with PTTD. There is a paucity of research surrounding the management of PTTD [3, 19]. This is the first survey to being to understand current practices in what health professionals offer this population in NHS practice. It indicates that there is variation both within and between professional groups notably physiotherapy and podiatry. Study limitations include the limited representation of respondents from Wales, Scotland and Northern Ireland. It therefore remains unclear whether local practices and commissioning processes, which may differ between these countries, could impact on practice. Secondly, the distribution of the survey was entirely online and via memberships of professional groups. This may therefore be a source of selection bias. Finally, the survey only explored current practices within the NHS. Many patients with PTTD may be seen in private practice. It therefore remains unclear whether there is a difference in NHS to private services for this population within the UK. Further study may be warranted not only to understand this difference but also to understand whether there are international differences in perspectives on how PTTD should be non‐surgically managed.

## 5 CONCLUSION

Management of patients with PTTD has been historically under‐researched. There are no guidelines on best practice in this area. This survey has demonstrated how clinicians assess, treat and evaluate management approaches for this population in NHS practice in the UK. These results will help to inform future research into the best management of this condition by providing a current overview of management to compare with.

## AUTHOR CONTRIBUTION

**Alison Miller**: Conceptualization; data curation; formal analysis; investigation; methodology; project administration; software; writing ‐ original draft; writing ‐ review & editing. **Toby Smith**: Conceptualization; methodology; supervision; writing ‐ review & editing. **Michael R. Backhouse**: Conceptualization; methodology; supervision; writing ‐ review & editing.

## CONFLICT OF INTEREST STATEMENT

No author declares a conflict of interest in relation to this paper.

## ETHICS STATEMENT

This study received ethical approval from the University of Warwick's Biomedical and Scientific Research Ethics Committee (BSREC 09/23–24).

## Supporting information

Supporting Information S1

## Data Availability

Data sharing is not applicable to this article as no new data were created or analysed in this study.
