# Supplementary material for: Non‐surgical management of posterior tibial tendon dysfunction‐ a UK survey
Source: J Foot Ankle Res. 2024 Jun 19;17(2):e12033. doi: 10.1002/jfa2.12033 (PMC11296709; doi:10.1002/jfa2.12033)
Supplement: Supplementary file 1 — Supporting Information S1 [file JFA2-17-e12033-s001.pdf]

Default Question Block

Posterior Tibial Tendon Dysfunction (PTTD) is an under-researched area. This questionnaire aims to explore the current practice in the non-surgical management of PTTD, across multiple professions working in the NHS. It will ask questions on diagnosis, treatment measures up to surgery, and outcome measures used. Following this we aim to publish the findings of this survey and plan future research to improve the evidence-base.
This questionnaire is voluntary and anonymous and we will not record any identifiable data.
The questionnaire should take approx 10 mins to complete.
Many thanks for your consideration. If you would like to complete the survey, please click through to the next page.

For further information please see our Participant Information Leaflet
Participant information leaflet survey pttd

Contact details alison.miller@warwick.ac.uk

- ○ Yes, I consent to completing this survey
- ○ No I do not wish to participate

Do you see and treat PTTD as part of your NHS practice?

- ○ No
- ○ Yes

What is your profession?

- ○ General Practitioner
- ○ Orthopaedic Surgeon
- ○ Physiotherapist
- ○ Podiatrist
- ○ Podiatric Surgeon
- ○ Rheumatologist
- ○ [ ] Other- please state

Please select which option best describes your main clinical service

- First contact practitioner
- General practice
- Integrated musculoskeletal
- Orthopaedic foot and ankle surgery
- Physiotherapy
- Podiatry
- Podiatric surgery
- Rheumatology
- Other- please state

Where is your service delivered?

- Community
- Primary care
- Secondary care
- Tertiary centre
- Other- please state

Where is your service located?

- England
- Northern Ireland
- Scotland
- Wales

How many people do you treat with PTTD each year?

- 1-5
- 6-15
- 16-25
- 26-50
- >50

Do you ever use imaging for these patients?

- ○ No
- ○ Yes

If yes, at what point would you consider this

- ☐ Following failed conservative management for 3 months or more
- ☐ For initial diagnosis
- ☐ To aid further management decisions
- ☐ To establish the state of the tendon
- ☐ To reassure the patient
- ☐ [ ] Other- please state

What modality of imaging would you choose initially?

- ○ Magnetic Resonance Imaging (MRI)
- ○ Ultrasound Scan (USS)
- ○ X-Ray
- ○ [ ] Other- please state

What treatment methods do you use routinely? (please move the toggle bar)

O- never use; 10-always use

	0	1	2	3	4	5	6	7	8	9	10	
Acupuncture												[ ]
Education												[ ]
Exercises- general (cardiovascular, lower limb)												[ ]
Exercises- specific to the foot												[ ]
Extracoporeal Shockwave Therapy (ESWT)												[ ]
Foot orthoses- custom made												[ ]
Foot orthoses- prefabricated												[ ]
Footwear advice												[ ]

O- never use; 10-always use

	0	1	2	3	4	5	6	7	8	9	10	
Injection- autologous blood												☐
Injection- corticosteroid												☐
Injection- plasma rich protein (PRP)												☐
Low level laser												☐
Splint/ brace												☐
Taping												☐
Therapeutic Ultrasound												☐
Weight loss advice												☐
Other- please state ☐												☐

Which specific foot exercises would you consider? (Tick all that Apply)

- ☐ Stretches
- ☐ Isometric inversion
- ☐ Eccentric
- ☐ Concentric
- ☐ Intrinsics
- ☐ Proprioception
- ☐ None
- ☐ ☐ Other- please state

Which type of general exercise would you use? (Tick all that apply)

- ☐ Cardiovascular exercise
- ☐ Kinetic chain exercises
- ☐ Hip exercises
- ☐ None
- ☐ ☐ Other- please state

11. Do you routinely use any specific outcome measures in your practice for patients

with PTTD? (tick all that apply)

- ☐ 5m walk test
- ☐ Foot Function Index (FFI)
- ☐ Short MSK Functional Assessment (SMFA)
- ☐ Single leg heel raise
- ☐ Manchester Oxford Foot and Ankle Questionnaire (MOXFQ)
- ☐ Musculoskeletal Health Questionnaire (MSK-HQ)
- ☐ Pain scale
- ☐ None
- ☐ ________ Other- please state

At what point would you consider onward referral for/ perform surgical intervention? (tick all that apply)

- ☐ Failure to manage symptoms with conservative management
- ☐ Fixed deformity
- ☐ Not applicable
- ☐ ________ Other- please state

Powered by Qualtrics
